# Growing Ignorance of COVID-19 Preventive Measures in Ethiopia: Experts' Perspective on the Need of Effective Health Communication Strategies

**DOI:** 10.4314/ejhs.v31i1.22

**Published:** 2021-01

**Authors:** Zewdneh Shewamene, Fisseha Shiferie, Engida Girma, Befikadu L Wubishet, Mizan Kiros, Atakelti Abraha, Abduljelil Reshad, Getachew Tiruneh, Benyam Worku, Eskedar Lemlemu, Rahel Belete Balkew

**Affiliations:** 1 Ethiopian Health Insurance Agency, Addis Ababa, Ethiopia; 2 Addis Continental Institute of Public Health, Addis Ababa, Ethiopia; 3 College of Health Sciences, Addis Ababa University, Addis Ababa, Ethiopia; 4 The University of Newcastle, Newcastle, Australia; 5 College of Social Sciences and Humanities, Debre Markos University, Ethiopia; 6 Independent Researcher, Addis Ababa, Ethiopia

**Keywords:** COVID-19, Prevention measures, Health communication, Ethiopia

## Abstract

Despite the recent surge of COVID-19 infections in Ethiopia, we are observing a profound ignorance of preventive measures by the general public and leaders at different levels. This is presenting considerable challenges in the effort to contain and control the pandemic. We believe that the current health communication approach implemented by the health authorities and media outlets need to be redesigned to bring a sustainable COVID-19 preventive behavior. The purpose of this perspective paper, therefore, is to stimulate discussions on effective health communication strategy to help the public persistently practice COVID-19 preventive measures over the long term. We undertook a series of discussions amongst the authors in order to synthesize individual viewpoints into ‘experts' perspective’ driven by our daily observations and our expertise in the health service research. In light of this, we suggested that an effective health communication strategy need to address context specific situations to avoid temptation to ignore the ramifications of this very serious pandemic. This strategy includes trying to make sense of daily reported COVID-19 cases, being highly selective regarding sources of information, and being sensitive and responsive to religious and cultural factors. The media, health professionals, and leaders need to teach us how to live with the pandemic informed by robust scientific sources.

## Introduction

**Figure 1:**
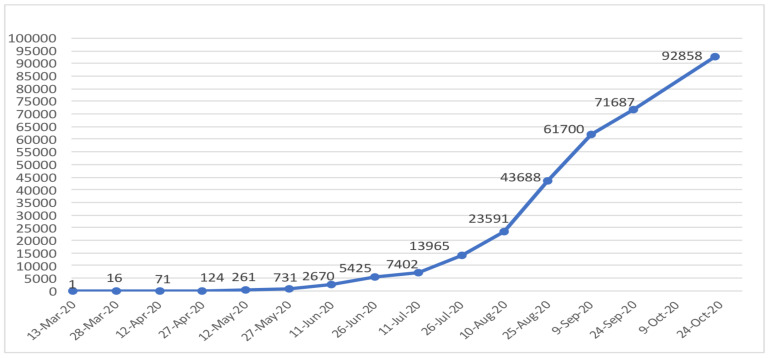
Confirmed COVID-19 cases in Ethiopia between March 13 and October 24, 2020. Source: Compiled by the authors using data from the Federal Ministry of Health, Ethiopia. The data presented in the figure refelects the evidence at the time of writing this perspective in October 2020. As COVID-19 is emerging, the data changes rapidly every day.

As of 31 October 2020, COVID-19 cases are increasing in Ethiopia steadily with 96,169 confirmed cases, 1,469 deaths and 52,517 recoveries (1) (Figure 1). It is also highly likely that there is a massive under detection of cases as the testing capacity is limited and more than 80% of the population live in rural areas with no easy access to testing.

Prior to the first confirmed case of the novel coronavirus in the African region, Ethiopia was among the 13 African countries that the World Health Organization (WHO) considered as a top priority for COVID-19 preparedness due to direct links or a high volume of travel to China (2). Since then, the government of Ethiopia has made massive preparedness efforts to contain a potential COVID-19 outbreak in collaboration with WHO and other partners (3,4).

After the first case was reported on the 13^th^ of March 2020, the government has put measures in place including declaring a state of emergency, implementing mass communication around social distancing, hand hygiene and wearing masks. The government implemented measures including banning public gatherings, closing schools and universities, and announcing the postponement of highly anticipated national elections previously scheduled for August 2020 (5). However, there were also some controversial decisions. Unlike most African counterparts, Ethiopia did not introduce a national lockdown. In addition, the Ethiopian Airlines continued its regular operation to China and other pandemic epicenter destinations until the end of March 2020 although many countries have grounded their flights (6,7).

**Ignorance of preventive measures amid surge in COVID-19 infections**: Despite the increasing number of daily reported cases, we are witnessing a growing public ignorance of the COVID-19 preventive measures. Increasing levels of negligence, and the circulation of myths and misinformation about the pandemic within the community complicate efforts to contain the crisis, resulting in a rise in the number of coronavirus cases and deaths. We are observing that some of these critical prevention measures against the pandemic that the community were exercising during the initial stage of the outbreak are gradually disappearing. For instance, physical distancing is not strictly implemented at high risk places including open marketplaces, religious institutions, and bus/train stops. The practice of physical distancing is becoming less common as people slip back into pre-COVID-19 habits and become fatigued with all the restrictions. In addition, access to hand washing and sanitizing facilities around congregational places that were readily accessible during the early panic stage of the outbreak are now disappearing. There were also other major events that diverted the publics' attention to other agendas including religious holidays, delay of the August 2020 election, and civil unrest in some regions of the country following the killing of a renowned artist.

**The need to revisit health information communication strategies**: Unfortunately, the current approach to delivering COVID-19 health information to the public is not optimal. For instance, messages disseminated from the Federal Ministry of Health and media on a daily basis are too brief and repetitive leading to information fatigue and negligence in adhering to restrictions. Daily reports from several media outlets and health authorities merely focused on the number of people infected, deaths, or recovered cases. This made the public pay more attention to the numbers than the inevitable consequences of the disease. Therefore, now is a critical time for local health authorities to rethink effective health information communication strategies to reduce ignorance and keep the public adherent to COVID-19 preventive measures. Here, we present some of the context specific issues that the current health information communication strategy should address to help sustain COVID-19 preventive behaviors in our community. We undertook a series of discussions amongst the authors in order to synthesize individual viewpoints into ‘experts' perspective’ driven by our daily observations and our expertise in the health service research.

**Make sense of reported numbers**: Numbers and mere statistics tend to dehumanize people and may not help us understand the physical and psychosocial suffering of infected individuals and their families. Reporting only the numbers is generally not effective in bringing about enduring behavioral change and in helping the public make sense of risks associated with the pandemic. We often are better motivated by the experiences of those who have experienced COVID-19. As such, presenting salient examples of the lived experience of infected and recovered individuals will humanize the daily reported numbers. This will also help the public easily relate with the afflicted or the deceased and make the pandemic real among the public. The seriousness of the risk posed by the virus will be accepted by the public when the impacts of the pandemic at the individual, family, and society levels are communicated. In turn, such an approach will help reduce public ignorance of the COVID-19 preventive measures implemented in efforts to prevent the spread of the virus and contain the pandemic.

**Increase awareness**: It is vital to ensure that our health information communication strategy is designed to improve basic knowledge and attitude towards COVID-19 to help the public engage in preventive behaviors. Despite efforts to promote public health awareness on COVID-19 preventive measures, adequate behavioral changes at all levels of the population in Ethiopia are yet to be achieved. We also have been witnessing denial and disbelief in accepting the fact that COVID-19 affects all people of color, race, and ages. This perception was particularly important in the beginning of the outbreak given that the virus took a relatively long time to be detected in Africa. As such, it is imperative to regularly provide updated health education to fill the huge gap in our understanding and misconceptions with regard to COVID-19. Extensive effort is vital to inform the public about the fact that the best way to fight the pandemic currently is through non-pharmaceutical interventions such as social distancing, hand washing/sanitizing, proper use of masks and avoiding face touching.

**Sensitive to religious and cultural issues**: The COVID-19 health information communication strategy needs to be sensitive and responsive to religious and cultural factors. Ethiopia is one of those conservatively religious countries in the world. Spiritual explanations of health issues with regard to attribution, intervention and coping strategies tend to be more widely accepted than the biomedical model, especially during challenging circumstances such as pandemics. In addition, for most Ethiopians, traditional and cultural medicine is the first line of healthcare. In effect, this may partly contribute to the observed ignorance of the recommended preventive measures. Although the participation of faith and traditional leaders is encouraging, it is crucial that our health communication strategy is designed to educate the public about the importance of preventive behaviors while allowing plurality in religious practices, traditional medicine, and cultural beliefs. It is also equally important to avoid denouncing statements related to the role of religious prospects and traditional medicine in COVID-19 prevention which may negatively impact the uptake of health education messages.

**Fight misinformation**: A further critical element of designing an effective COVID-19 health information communication strategy should be fighting misinformation surrounding the pandemic. The outbreak has been accompanied by a massive ‘infodemic’. Excessive volume of information about COVID-19 (including false prevention measures or cures) poses concerns for the public to distinguish fact from fiction, and for government agencies to conduct evidence-based policy-making (8). The misinformation around the cause, symptoms, and mode of transmission has been overwhelming. We have witnessed that misleading and contradicting information is not only coming from the internet or social media, but also from political leaders and health authorities. For example, a press briefing communicated by the Ministry of Health and Ministry of Science and Innovation on the discovery of a potential traditional treatment for COVID-19 has spurred tremendous hope in the public and may lead to ignorance of existing preventive measures. Although all efforts in the search for treatment and vaccine are praised and appreciated, such information need to be communicated to the public in a more responsible and cautious approach. Overall, addressing misinformation in our communication strategy is exceedingly important to improve public confidence and trust in the effectiveness of COVID-19 preventive measures.

**Promote resilience**: Finally, a health information communication strategy intended to promote resilience and adaptation to the ‘new normal’ will have an indispensable role in enhancing adherence to COVID-19 prevention, and also in educating the public on how to live with the pandemic in the long term. The progressive decline in people's motivation to perform COVID-19 preventive behaviors might be partly attributed to the fact that there is a need to consistently apply these measures for several months, if not years, until the pandemic is eradicated or effective vaccine or treatment options are available. It is necessary for individuals and families to change their way of life, even though it is particularly challenging for interdependent societies. Our daily life needs to incorporate wearing face masks, physical distancing, proper handwashing, applying hand sanitizers, and other infection prevention measures. As such, developing our communication strategy to advocate sustainably of these behaviors will be important to contain the pandemic. In light of this, effective health communication needs to address context specific situations to avoid temptation to ignore the ramifications of this very serious pandemic. The information we get from the media, health authorities, and leaders should help us to carefully appraise the seriousness of the problem without panicing.

In conclusion, the coronavirus infection is surging at an alarming rate in Ethiopia. This has given rise to several challenges on an unprecedented scale ranging from public health emergencies to political and societal problems, and a significant disruption of the economy. However, more alarming than the current surge of positive cases is the progressive ignorance of the public to perform preventive behaviors. Effective and innovative COVID-19 health information communication strategies are imperative to encourage and sustain adherence to preventive behaviors, in efforts to flatten the case and death curves and mitigate the impact of the COVID-19 pandemic in Ethiopia.
